# Infant Botulism Caused by *Clostridium baratii* Type F — Iowa, 2013

**Published:** 2015-04-17

**Authors:** Amaran Moodley, Patricia Quinlisk, Ann Garvey, Nicholas Kalas, Jason R. Barash, Jessica M. Khouri

**Affiliations:** 1 Division of Infectious Diseases, Department of Pediatrics, Blank Children’s Hospital, Des Moines, Iowa; 2 Division of Acute Disease Prevention, Emergency Response, and Environmental Health, Iowa Department of Public Health; 3 Infant Botulism Treatment and Prevention Program, California Department of Public Health

In June 2013, a male newborn aged 9 days (delivered after a full-term pregnancy) was brought to a hospital emergency department with a 2-day history of constipation, fussiness, and poor feeding. The mother reported her son’s symptoms as excessive crying, reluctance to suck, and difficulty in swallowing milk. Within hours of arrival, the infant became less responsive and “floppy,” and was intubated for respiratory failure. Infant botulism was suspected and Botulism Immune Globulin Intravenous (Human) (BIG-IV), licensed for the treatment of infant botulism types A and B, was administered on hospital day 2. Results of preliminary stool studies were reported positive for botulinum toxin type F on hospital day 3. *Clostridium baratii* type F was subsequently isolated in stool culture.

National experience with type F botulism in newborns and infants indicates that rapid clinical improvement could occur even without the administration of anti-type F antitoxin. However, 3 days after treatment with BIG-IV the newborn continued to require ventilator support and showed no signs of clinical improvement. On hospital day 6, equine-derived botulism antitoxin heptavalent (A-G) (BAT) was administered to the boy, despite the limited experience reported for its use in pediatric cases. This is the second newborn treated with BAT in the United States; the first was treated in 2008 in Colorado (1).

Within 24 hours of BAT treatment, spontaneous movements of the newborn’s extremities increased. On hospital day 8 the endotracheal tube was removed. By the following day, the boy could tolerate oral feedings, had regained muscle tone and strength in his extremities, and had normal pupillary responses. The only adverse event associated with BAT treatment was an intermittent, low-grade fever that developed within 1 hour of BAT administration and lasted 72 hours. Blood, urine, stool, and cerebrospinal fluid bacterial cultures were otherwise negative. Contrast magnetic resonance imaging of his brain showed normal findings, and cerebrospinal fluid studies for herpes simplex virus and enterovirus also were negative. The newborn was discharged on hospital day 12. At the 2-week follow-up examination, his mother reported he was doing well: taking 100% of his feedings orally, exhibiting no residual weakness, and having normal bowel movements.

The parents reported feeding the newborn ready-to-feed and powdered formula from the same brand. No other solid or liquid foods or homeopathic remedies or supplements were given before symptom onset. No classic risk factors for infant botulism were reported, such as exposure to honey or soil. The parents reported strong winds and minor construction in the area surrounding their home. Pets present in the home included cats, turtles, fish, geckos, sugar gliders, and a mouse.

Environmental samples were collected from 1) feces from all animals in the home, 2) food and water from the turtle enclosure, 3) dust from the vacuum cleaner bag and the windowsill and ceiling fan closest to where the child slept, and 4) potting soil from the only indoor plant in the home. Although *Clostridium* species were isolated in several of the samples, none produced botulinum toxin.

Through 2012, only 13 cases of *C. baratii* type F infant botulism have been reported in the United States; this is the third confirmed case in Iowa. Extensive investigations for an environmental source of toxigenic *C. baratii* have been undertaken, including for all three cases in Iowa (2). Unlike typical infant botulism caused by *C. botulinum* (3), no source has been identified and prevention strategies remain unknown for *C. baratii*. While *C. baratii* infant botulism remains a rarely diagnosed disease, health care providers should maintain a high index of suspicion especially in very young infants who present with new onset floppiness or progressive respiratory failure.
